# Supplementary material for: Vascularisation in Deep Endometriosis: A Systematic Review with Narrative Outcomes
Source: Cells. 2023 May 5;12(9):1318. doi: 10.3390/cells12091318 (PMC10177118; doi:10.3390/cells12091318)
Supplement: Supplementary file 1 [file cells-12-01318-s001.zip › cells-2346610-supplementary.pdf]

**Supplementary Table S1 – Search Strategy**

| Set | Search Statement |
|---|---|
| **1** | (Deep Infiltrating Endometriosis or Deep Endometriosis or Bowel endometriosis or Colonic endometriosis or colon endometriosis or rectal endometriosis or rectovaginal endometriosis or rectal-vaginal endometriosis or (endometriosis and penetrating) or (Endometriosis and Deep) or (endometriosis and Rectum) or rectosigmoid endometriosis or Recto-Sigmoid endometriosis or (sigmoid and endometriosis)).mp. [mp=title, book title, abstract, original title, name of substance word, subject heading word, floating sub-heading word, keyword heading word, organism supplementary concept word, protocol supplementary concept word, rare disease supplementary concept word, unique identifier, synonyms, population supplementary concept word, anatomy supplementary concept word] |
| **2** | (Vasculari*ation or Neovasculari*ation or Angiogenesis or "blood supply" or vasculogenesis or inosculation).mp. or Blood Vessels/ or Pathologic Neovascularization.mp. or (Neovascularization, Physiologic/ or Neovascularization, Pathologic/) or (Angiogenesis, Pathologic or Angiogenesis, Pathological or Angiogenesis, Physiologic or Angiogenesis, Physiological or Pathologic Angiogenesis or Pathological Angiogenesis or Physiologic Angiogenesis or Physiological Angiogenesis).mp. [mp=title, book title, abstract, original title, name of substance word, subject heading word, floating sub-heading word, keyword heading word, organism supplementary concept word, protocol supplementary concept word, rare disease supplementary concept word, unique identifier, synonyms, population supplementary concept word, anatomy supplementary concept word] |
| **3** | 1 and 2 |
